# Dysphagia and Dysarthria in Neurodegenerative Diseases: A Multisystem Network Approach to Assessment and Management

**DOI:** 10.3390/audiolres16010009

**Published:** 2026-01-12

**Authors:** Maria Luisa Fiorella, Luca Ballini, Valentina Lavermicocca, Maria Sterpeta Ragno, Domenico A. Restivo, Rosario Marchese-Ragona

**Affiliations:** 1 Otolaryngology Unit, Department of Biomedical Sciences, Neuroscience and Sensory Organs, University of Bari “Aldo Moro”, 70121 Bari, Italy; mariaragno.med@gmail.com; 2 Otolaryngology Section, Department of Neurosciences, University of Padova, 35122 Padova, Italy; luca.ballini@aopd.veneto.it (L.B.); rosario.marcheseragona@unipd.it (R.M.-R.); 3 Physical and Rehabilitation Medicine Unit, Department of Biomedical Sciences, Neuroscience and Sensory Organs, University of Bari “Aldo Moro”, 70121 Bari, Italy; valentina.lavermicocca@uniba.it; 4 Physical Medicine and Rehabilitation Unit, Department of Clinical and Experimental Medicine, University of Messina, 98122 Messina, Italy; domenicoantonio.restivo@unime.it

**Keywords:** dysphagia, dysarthria, neurodegenerative diseases, brainstem, neural networks, multidisciplinary management

## Abstract

Dysphagia and dysarthria are common, co-occurring manifestations in neurodegenerative diseases, resulting from damage to distributed neural networks involving cortical, subcortical, cerebellar, and brainstem regions. These disorders profoundly affect patient health and quality of life through complex sensorimotor impairments. **Objective**: The aims was to provide a comprehensive, evidence-based review of the neuroanatomical substrates, pathophysiology, diagnostic approaches, and management strategies for dysphagia and dysarthria in neurodegenerative diseases with emphasis on their multisystem nature and integrated treatment approaches. **Methods**: A narrative literature review was conducted using PubMed, Scopus, and Web of Science databases (2000–2024), focusing on Parkinson’s disease (PD), amyotrophic lateral sclerosis (ALS), progressive supranuclear palsy (PSP), and multiple system atrophy (MSA). Search terms included “dysphagia”, “dysarthria”, “neurodegenerative diseases”, “neural networks”, “swallowing control” and “speech production.” Studies on neuroanatomy, pathophysiology, diagnostic tools, and therapeutic interventions were included. **Results**: Contemporary neuroscience demonstrates that swallowing and speech control involve extensive neural networks beyond the brainstem, including bilateral sensorimotor cortex, insula, cingulate gyrus, basal ganglia, and cerebellum. Disease-specific patterns reflect multisystem involvement: PD affects basal ganglia and multiple brainstem nuclei; ALS involves cortical and brainstem motor neurons; MSA causes widespread autonomic and motor degeneration; PSP produces tau-related damage across multiple brain regions. Diagnostic approaches combining fiberoptic endoscopic evaluation, videofluoroscopy, acoustic analysis, and neuroimaging enable precise characterization. Management requires multidisciplinary Integrated teams implementing coordinated speech-swallowing therapy, pharmacological interventions, and assistive technologies. **Conclusions**: Dysphagia and dysarthria in neurodegenerative diseases result from multifocal brain damage affecting distributed neural networks. Understanding this multisystem pathophysiology enables more effective integrated assessment and treatment approaches, enhancing patient outcomes and quality of life.

## 1. Introduction

Swallowing and speech represent highly sophisticated neuromotor functions requiring coordinated activation of distributed neural networks spanning cortical, subcortical, cerebellar, and brainstem regions [1,2]. Contemporary neuroscience has established that these functions are controlled not solely by brainstem centers, but by extensive bilateral cortical and subcortical networks with critical integrative roles in the insula, sensorimotor cortex, and basal ganglia [3,4,5].

Neurodegenerative diseases frequently compromise both systems simultaneously through multifocal brain damage, resulting in dysphagia (swallowing dysfunction) and dysarthria (motor speech disorder) [1,3,6]. The co-occurrence of these disorders reflects their overlapping neural substrates and shared pathophysiological mechanisms involving multiple brain regions [2,3,4].

This review examines current evidence on the distributed neural networks controlling swallowing and speech, discusses disease-specific patterns of multisystem involvement in Parkinson’s disease (PD), amyotrophic lateral sclerosis (ALS), progressive supranuclear palsy (PSP), and multiple system atrophy (MSA), and analyzes integrated management strategies addressing both functions within a comprehensive multidisciplinary framework.

## 2. Methods

### Search Strategy and Selection Criteria

A comprehensive literature search was conducted using PubMed, Scopus, and Web of Science databases for publications from January 2000 to December 2024. The search strategy combined MeSH terms and keywords including: “dysphagia”, “dysarthria”, “deglutition disorders”, “speech disorders”, “Parkinson’s disease”, “amyotrophic lateral sclerosis”, “progressive supranuclear palsy”, “multiple system atrophy”, “neurodegenerative diseases”, “neural networks”, “Brainstem Neurodegeneration”, “brain connectivity”, “swallowing control” and “speech production.”

**Inclusion criteria:**

- Studies on swallowing and/or speech disorders in neurodegenerative diseases.
- Research on neuroanatomical substrates and neural networks.
- Clinical trials and observational studies on diagnostic and therapeutic interventions.
- Systematic reviews and meta-analyses.
- English language publications.

**Exclusion criteria:**

- Case series with fewer than 5 patients.
- Studies on non-neurodegenerative causes of dysphagia/dysarthria.
- Animal studies without clinical relevance.

Additional references were identified through citation tracking of key articles and recent consensus guidelines. One should note that this is a narrative commentary and there was no formal risk of bias assessment.

## 3. Neuroanatomical Substrate: A Distributed Network Perspective

### 3.1. The Multisystem Neural Network for Swallowing Control

Contemporary neuroimaging and electrophysiological research demonstrates that swallowing control involves a distributed bilateral network far exceeding brainstem centers [3,4,5], as detailed in Table 1.

**Cortical and Subcortical Structures:**

The primary sensorimotor cortex shows bilateral activation with left hemisphere dominance [4,5], while the insula, also left-lateralized, serves as the primary integrative hub [3,4]. The cingulate gyrus contributes to volitional control and sensory integration, the supplementary motor area to movement preparation and sequencing, and the parietal lobules to oral and pharyngeal sensory processing. Subcortically, the thalamus relays sensory information and modulates motor output, while the basal ganglia regulate initiation, timing, and amplitude through dopaminergic circuits [6,7].

**Cerebellar and Brainstem Centers:**

The cerebellum coordinates temporal sequencing, muscle force, and movement precision. Brainstem nuclei form the core effector system: the Nucleus Tractus Solitarius (NTS) acts as central pattern generator [1,3], the Nucleus Ambiguus (NA) innervates pharyngeal and laryngeal muscles [1,2], and the dorsal motor nucleus of vagus controls esophageal motility. Cranial nerve nuclei (XII, VII, V) govern tongue, perioral, and jaw movements, respectively.

Functional connectivity studies reveal dynamic connections between sensorimotor cortex and the broader swallowing network, with connectivity amplified during swallowing tasks [3,4,5].

### 3.2. The Multisystem Neural Network for Speech Production

Speech production relies on analogous distributed networks, as shown in Table 1.

**Cortical and Subcortical Structures:**

The primary motor cortex contains somatotopic representations for articulators (face, lips, tongue, larynx). Broca’s area, left-dominant, handles motor programming, while premotor regions manage planning and sequencing. The insula coordinates articulatory execution. Basal ganglia modulate initiation, amplitude, and prosody [6,8,9]; the thalamus provides command relay and feedback regulation.

**Cerebellar and Brainstem Centers:**

Cerebellar contributions extend to rhythm and prosodic control. The nucleus ambiguus governs phonation, while hypoglossal, facial, and trigeminal nuclei direct articulator movements. Respiratory centers ensure breathing-phonation synchronization [5,10].

### 3.3. Convergent Pathways and Shared Mechanisms

The extensive anatomical and functional overlap explains frequent co-impairment in neurological diseases [1,3,9]: shared cortical planning regions (insula, sensorimotor cortex, supplementary motor area), common brainstem effector nuclei (ambiguus, hypoglossal, facial), integrated respiratory control [5,10], and dopaminergic basal ganglia modulation [6,8].

## 4. Pathophysiology: Multisystem Damage Patterns in Specific Diseases

### 4.1. Parkinson’s Disease: Beyond Substantia Nigra

PD pathology extends far beyond classic substantia nigra pars compacta degeneration, involving multiple brain regions that affect both swallowing and speech [6,8,11,12], as summarized in Table 2.

**Multisystem Involvement:**

- **Basal ganglia:** Dopaminergic deficiency impairs movement initiation, amplitude scaling, and sequential coordination.
- **Dorsal motor nucleus of vagus:** Neuronal loss contributes to esophageal dysmotility and autonomic dysfunction.
- **Locus coeruleus:** Noradrenergic depletion affects arousal and motor control.
- **Raphe nuclei**: Serotonergic dysfunction impacts motor and autonomic regulation.
- **Cortical regions:** Alpha-synuclein pathology in later stages affects cortical motor planning.

**Clinical Manifestations:**

*Dysphagia:* Bradykinesia and rigidity compromise oral phase control, delayed swallow initiation, and reduced pharyngeal coordination [8,11]. Impaired laryngeal closure increases aspiration risk. Autonomic dysfunction causes sialorrhea and gastroparesis. Cognitive impairment may affect volitional swallowing control.

*Dysarthria:* Hypokinetic dysarthria predominates (10–20% show mixed hypokinetic-hyperkinetic patterns), characterized by reduced loudness, monotone speech, imprecise articulation, and variable speech rate [9,13,14,15]. The same basal ganglia dysfunction affecting swallowing compromises speech amplitude and prosody.

### 4.2. Amyotrophic Lateral Sclerosis: Cortical and Brainstem Motor System Degeneration

ALS involves progressive degeneration of both upper motor neurons (cortical) and lower motor neurons (brainstem and spinal cord), producing distinctive mixed pathology [2,16].

**Dual Motor System Involvement:**

- **Cortical motor neurons:** Degeneration of pyramidal cells in primary motor cortex affects corticobulbar pathways.
- **Brainstem motor nuclei:** Lower motor neuron loss in hypoglossal, facial, trigeminal, and nucleus ambiguus produces weakness and atrophy.
- **Corticobulbar tracts:** White matter degeneration disrupts upper motor neuron control.

**Clinical Manifestations:**

*Dysphagia*: Combined upper and lower motor neuron involvement affects all swallowing phases [2,15]. Tongue weakness impairs bolus formation, pharyngeal weakness delays swallow initiation, and cricopharyngeal dysfunction prevents efficient bolus transit. Progressive bulbar weakness dramatically increases aspiration risk.

*Dysarthria*: Mixed spastic-flaccid dysarthria combines upper motor neuron features (slow, effortful speech with harsh voice) and lower motor neuron characteristics (breathy voice, imprecise articulation, nasal emission) [9,13,14,15]. The same cortical and brainstem motor neuron loss affects both functions, progressing to anarthria.

### 4.3. Progressive Supranuclear Palsy: Widespread Tau-Related Pathology

PSP involves tau protein accumulation affecting multiple brain regions beyond the brainstem [1,8]:

**Multifocal Degeneration:**

- **Brainstem:** Substantia nigra, superior colliculus, periaqueductal gray, and reticular formation.
- **Basal ganglia:** Globus pallidus and striatum degeneration.
- **Frontal cortex:** Tau pathology in premotor and prefrontal regions.
- **Cerebellum:** Dentate nucleus involvement.

**Clinical Manifestations:**

*Dysphagia:* Delayed pharyngeal swallow initiation, laryngeal rigidity, and loss of oral-pharyngeal coordination reflect widespread motor system involvement [1,8]. Supranuclear gaze palsy may affect visual coordination during eating.

*Dysarthria:* Spastic-ataxic mixed dysarthria with harsh voice, reduced loudness, imprecise articulation, and prosodic disturbances [13,14,16]. Speech difficulties reflect the same rigidity and coordination deficits affecting swallowing.

### 4.4. Multiple System Atrophy: Autonomic and Motor System Degeneration

MSA involves widespread alpha-synuclein accumulation in oligodendrocytes affecting multiple systems [1,17]:

**Extensive Neurodegeneration:**

- **Brainstem:** Pontine nuclei, inferior olives, nucleus ambiguous.
- **Cerebellum:** Purkinje cell loss and cerebellar pathway degeneration.
- **Basal ganglia:** Striatal degeneration (particularly putamen).
- **Autonomic centers:** Intermediolateral cell column and autonomic nuclei.

**Clinical Manifestations:**

*Dysphagia:* Brainstem and nucleus ambiguus degeneration causes severe pharyngeal coordination deficits and laryngeal dysfunction [1,16]. Delayed swallow initiation and compromised airway protection increase aspiration risk. Autonomic dysfunction may contribute to sialorrhea and gastroparesis.

*Dysarthria:* Ataxic dysarthria with irregular articulatory breakdowns, excess and equal stress, and irregular rhythm reflecting cerebellar pathway involvement [13,14,16]. Some patients show mixed hypokinetic-ataxic features.

## 5. Assessment and Diagnosis

### 5.1. Integrated Assessment Framework

Comprehensive evaluation must acknowledge the interconnected nature of dysphagia and dysarthria, with protocols addressing both functions within a multidisciplinary framework [18,19]. Table 3 summarizes the diagnostic tools available.

**Initial Screening:**

- **Dysphagia screening:** Questionnaires assessing coughing/choking frequency, saliva management, and respiratory complications, followed by water-swallowing tests and multi-consistency protocols [8,11,18].
- **Speech screening:** Perceptual assessment using Mayo Clinic classification, evaluating respiratory, phonatory, articulatory, resonance, and prosodic subsystems [13,14].
- **Cognitive screening:** Montreal Cognitive Assessment (MoCA) and Frontal Assessment Battery (FAB) to identify cognitive factors affecting both functions.

### 5.2. Swallowing Assessment

**Clinical Evaluation:**

- Cranial nerve examination.
- Oral mechanism assessment.
- Saliva management observation.
- Clinical swallowing evaluation with multiple consistencies.

**Instrumental Evaluation:**

- **Videofluoroscopy (VFS):** Dynamic visualization of all swallowing phases, assessing timing, coordination, and aspiration/penetration across multiple consistencies [20].
- **Fiberoptic Endoscopic Evaluation of Swallowing (FEES):** Direct visualization of pharynx and larynx, identifying structural abnormalities, absent or reduced reflexes, motility impairment and aspiration/penetration [20,21].
- **High-Resolution Manometry (HRM):** Pharyngeal and esophageal pressure assessment, particularly valuable for esophageal dysfunction [2,3].
- **Electromyography (EMG):** Muscle activation patterns during swallowing, identifying targets for botulinum toxin therapy [2,7].

### 5.3. Speech Assessment

**Clinical Evaluation:**

- Motor speech mechanism examination (strength, range, speed, coordination, symmetry).
- Perceptual speech assessment using Frenchay Dysarthria Assessment-2 (FDA-2) [22].
- Dysarthria subtype classification [13,14].
- Screening for Aphasia in Neurodegeneration (SAND) to differentiate motor speech from language disorders [23].

**Instrumental Analysis:**

*Acoustic Analysis:* Objective measurement using software (Praat, Computerized Speech Lab) for [19,24,25,26,27]:

- Fundamental frequency (F0) and variability (pitch monotony vs. pitch breaks).
- Jitter and shimmer representing voice instability.
- Voice Onset Time (VOT) critical in differentiating between spastic and flaccid dysarthria.
- Speech rate measured in syllables per second and diadochokinetic (DDK) rates using alternating motion rates (AMRs) and sequential motion rates (SMRs) for motor planning evaluation.
- Vowel Space Area (VSA) for intelligibility assessment.

*Aerodynamic Assessment:* Evaluation using Phonatory Aerodynamic System (PAS) for subglottic pressure and airflow during phonation [19,28].

Acoustic and aerodynamic analyses represent promising tools for early diagnosis, differential diagnosis, and monitoring of neurodegenerative diseases affecting speech and voice. Quantified vocal biomarkers enable objective tracking of disease progression and treatment response.

### 5.4. Neurophysiological and Neuroimaging Assessment

**Electrophysiological Testing:**

- **Laryngeal EMG:** Assesses neuromuscular transmission for both swallowing and speech functions [2,15]. By quantifying parameters such as motor unit recruitment, spontaneous activity, and reinnervation patterns, laryngeal EMG provides critical insights into the nature and severity of neurogenic involvement.
- **Transcranial Magnetic Stimulation (TMS):** Provides noninvasive evaluation of corticobulbar excitability and conduction time within motor pathways controlling speech and swallowing [19].

**Neuroimaging:**

- **Structural MRI:** Reveals atrophy patterns in relevant brain regions [1,4].
- **Diffusion Tensor Imaging (DTI):** Detects microstructural alterations in corticobulbar tracts [5].
- **Functional MRI (fMRI):** Identifies specific activation patterns during speech and swallowing tasks, highlighting dynamic engagement of cortical and subcortical regions [4,5].
- **PET Imaging:** Shows hypometabolism in speech-swallowing motor areas, elucidating functional impact of neurodegenerative processes [5].

**Cognitive and Linguistic Assessment:**

Cognitive and linguistic deficits may both mimic and mask motor speech disorders, making differential diagnosis particularly challenging. A structured comprehensive assessment is therefore essential:

- **Montreal Cognitive Assessment (MoCA):** Provides a sensitive measure of global cognitive function, including attention, memory, executive functioning, language, and visuospatial abilities influencing both swallowing safety and communication [23].
- **Frontal Assessment Battery (FAB):** Evaluates executive dysfunction, probing conceptualization, mental flexibility, inhibitory control, and motor programming.
- **Screening for Aphasia in Neurodegeneration (SAND):** Differentiates motor speech impairments from primary language disorders, identifying subtle aphasic features that may co-occur with or mimic dysarthria [23].

## 6. Management and Treatment Strategies

### 6.1. Multidisciplinary Team Approach

Given the multisystem nature of these disorders, effective management requires coordinated multidisciplinary teams including otolaryngologists, neurologists, speech-language pathologists, physiatrists, nutritionists, gastroenterologists, radiologists, and rehabilitation specialists [6,11,29,30]. Table 4 summarizes evidence-based management strategies.

### 6.2. Pharmacological Interventions

**Botulinum Toxin:** Effective for cricopharyngeal dysfunction in dysphagia and laryngeal spasticity in dysarthria [2,7,31,32,33,34]. Approximately 75% of patients show improvement, with effects lasting 4–6 months. Success rates vary by disease (higher in PD, lower in ALS).

**Disease-Modifying Medications:**

- Levodopa optimization in PD improves both swallowing and speech function [8,11].
- Cholinesterase inhibitors for cognitive aspects affecting swallowing control.
- Antispasticity agents (baclofen, tizanidine) in ALS [35].

**Sensory Enhancement:** Emerging evidence for capsaicin, piperine, and menthol to improve swallowing through enhanced sensory input and neuroplasticity.

### 6.3. Behavioral and Rehabilitative Interventions

**Coordinated Speech-Swallowing Therapy:**

*Dysphagia Interventions [6,11,36]:*

- **Compensatory strategies:** Postural adjustments (chin tuck, head rotation), modified bites/sips.
- **Rehabilitative exercises:** Mendelsohn maneuver, effortful swallow, supraglottic swallow, tongue strengthening.
- **Sensory techniques:** Thermal-tactile stimulation, taste/texture modifications.
- **Respiratory coordination:** Breath-hold techniques, voluntary cough training.

*Dysarthria Interventions [29,30]:*

- **Lee Silverman Voice Treatment (LSVT LOUD):** Evidence-based for PD, enhancing vocal intensity.
- **Pitch Limiting Voice Treatment (PLVT):** Targets phonation without pitch elevation [28].
- **Articulatory therapy:** Exaggerated articulation, rate control, pausing strategies.
- **Respiratory training:** Inspiratory/expiratory muscle strength training [5,10].
- **Prosodic training:** Pitch variation, stress patterns, rhythm exercises.

*Unified Approaches [6,29]:*

- Laryngeal strengthening exercises benefit both vocal fold closure and airway protection.
- Respiratory coordination training enhances both functions.
- Neuromuscular electrical stimulation (NMES) may improve pharyngeal function [37,38].

### 6.4. Neuromodulation Techniques

**Non-invasive Brain Stimulation [36,37]:**

- Repetitive transcranial magnetic stimulation (rTMS).
- Transcranial direct current stimulation (tDCS).
- Pharyngeal electrical stimulation (PES).

These techniques show promise for enhancing cortical excitability and motor learning in rehabilitation.

### 6.5. Compensatory Strategies and Assistive Technology

**Dietary Modifications:**

- Texture modification following IDDSI framework.
- Liquid thickening to reduce aspiration risk.
- Eating Assessment Tool-10 (EAT-10) for risk identification [38].

**Augmentative and Alternative Communication (AAC) [19,29]:**

- **Low-tech:** Communication boards, alphabet cards, writing.
- **High-tech:** Speech-generating devices, smartphone applications, eye-tracking systems.
- **Voice banking:** Preserves patient’s natural voice for future AAC use.
- **Message banking:** Records phrases in natural speech.
- **Biofeedback Systems:** Real-time visual/auditory feedback for training [19,27].

### 6.6. Nutritional Support

**Short-term Enteral Feeding:**

- Nasogastric (NG), nasoduodenal (ND), or orogastric tubes (<4–6 weeks).
- Parenteral nutrition when enteral access not feasible.

**Long-term Enteral Access [38,39]:**

- Percutaneous Endoscopic Gastrostomy (PEG): Local anesthesia with sedation.
- Radiologically Inserted Gastrostomy (RIG): Alternative under radiological guidance.
- PEG-J: Extension to jejunum for reflux or medication delivery (DUODOPA in PD).

### 6.7. Interventional Procedures

**Cricopharyngeal Dysfunction Management [2,31,32,33,34]:**

*Endoscopic Dilatation:* Balloon or tapered dilators under sedation, useful for fibrotic stenosis.

*Botulinum Toxin Injection:* 75% success rate overall, higher in PD. EMG-guided or endoscopic injection. Effects begin 24–72 h, lasting 4–6 months. Low complication rates.

*Surgical Cricopharyngeal Myotomy:* 75% average success rate. Endoscopic approach reduces complications versus open technique [32].

### 6.8. Airway Protection

**Tracheostomy:** For severe aspiration with inability to protect airway. Allows phonation with appropriate valves [15,29].

**Laryngeal Procedures:**

- **Injection laryngoplasty:** Improves glottic closure, reduces aspiration.
- **Functional laryngeal closure:** Separates respiratory and digestive tracts in severe refractory aspiration [15,29].

### 6.9. Emerging Therapies

**Gene Therapy:** Shows promise for neurodegenerative diseases including PD, ALS, though clinical applications remain experimental [35].

**Stem Cell Therapy:** Demonstrates potential in preclinical studies, requiring further clinical translation [40].

**Table 4:** Management Strategies Evidence Summary for Dysphagia and Dysarthria.

Intervention	Dysphagia Application	Dysarthria Application	Evidence Level	Disease-Specific Considerations
**Pharmacological**				
Botulinum Toxin	Cricopharyngeal dysfunction (75% success)	Laryngeal dystonia, spasticity	Level II	Better outcomes in PD vs ALS
Levodopa	Improves swallow timing in PD	Increases vocal loudness in PD	Level I (PD)	Optimize ON state for meals/speech
Cholinesterase Inhibitors	May improve swallow Cognition	Limited effect	Level III	Consider in PD dementia
Antispasticity Agents	Limited evidence	Reduces spasticity in ALS	Level II (ALS)	Baclofen, tizanidine
Sensory Enhancers	Capsaicin, menthol improve trigger	Not applicable	Level II	Short-term effects
**Behavioral/Rehabilitative**				
Compensatory Strategies	Chin tuck, head rotation, Pacing	Rate control, clear speech	Level I	Disease-specific modifications
Strengthening Exercises	Tongue, pharyngeal Exercises	LSVT LOUD, PLVT	Level I	LSVT proven in PD
Sensory Techniques	Thermal-tactile, taste/texture	Not applicable	Level II	Useful in sensory deficits
Respiratory Training	Supraglottic swallow, Cough	EMST, IMST	Level I	Benefits both functions
**Neuromodulation**				
rTMS	Cortical excitability Enhancement	Limited studies	Level II	Research setting
tDCS	Swallow motor learning	Speech motor learning	Level III	Emerging evidence
PES	Pharyngeal stimulation	Not applicable	Level II	Post-stroke mainly
NMES	Pharyngeal strengthening	Limited application	Level III	Controversial
**Assistive Technology**				
Dietary Modification	Texture modification, Thickening	Not applicable	Level I	IDDSI framework
AAC	Not applicable	Low-tech to high- tech devices	Level I	Early implementation in ALS
Voice Banking	Not applicable	Preserves natural voice	Level II	Critical in ALS/PSP
Biofeedback	sEMG swallow training	Visual/auditory speech feedback	Level II	Adjunct to therapy
**Nutritional Support**				
Enteral Feeding (NG/PEG)	Severe dysphagia, Aspiration	Not applicable	Level I	PEG timing crucial in ALS [35]
**Surgical/Interventional**				
Cricopharyngeal Myotomy	UES dysfunction (75% success)	Not applicable	Level II	Consider after BTX failure
Injection Laryngoplasty	Glottic insufficiency, Aspiration	Improves voice quality	Level II	Temporary in progressive disease
Tracheostomy	Severe aspiration, airway Protection	Allows phonation with valve	Level I	QOL consideration

## 7. Discussion

This review emphasizes that dysphagia and dysarthria in neurodegenerative diseases result from damage to distributed neural networks spanning cortical, subcortical, cerebellar, and brainstem regions, rather than isolated brainstem pathology. This multisystem perspective has important clinical implications:

**Pathophysiological Understanding:** Recognition that PD affects basal ganglia and multiple brainstem nuclei, ALS involves cortical and brainstem motor systems, PSP produces widespread tau-related damage, and MSA causes extensive autonomic and motor degeneration enables more accurate prognostic assessment and targeted interventions.

**Assessment Strategies:** Comprehensive evaluation must incorporate tools assessing multiple levels of the neural network, including neuroimaging of cortical and subcortical structures, electrophysiological testing of corticobulbar pathways, and functional assessments of both swallowing and speech simultaneously.

**Treatment Approaches:** Understanding multisystem involvement guides selection of interventions targeting specific network components. Pharmacological treatments address specific neurotransmitter or peripheral mechanisms. Behavioral therapies leverage neuroplasticity across the distributed network. Neuromodulation techniques target cortical excitability.

**Integrated Care:** The overlapping neural substrates mandate unified assessment and management approaches delivered by multidisciplinary teams, addressing both functions concurrently rather than in isolation.

**Future Directions:** Emerging therapies including gene therapy, stem cell interventions, and advanced neuromodulation techniques targeting specific network components hold promise but require rigorous clinical validation.

## 8. Conclusions

Dysphagia and dysarthria in neurodegenerative diseases are multifactorial conditions resulting from damage to distributed neural networks involving cortical, subcortical, cerebellar, and brainstem regions. Understanding this multisystem pathophysiology is essential for accurate diagnosis and effective management.

Key clinical implications include:

- Early and iterative evaluation using comprehensive assessment protocols acknowledging interconnected neural networks.
- Integrated multidisciplinary management approaches addressing both swallowing and speech simultaneously.
- Disease-specific treatment strategies targeting specific patterns of network involvement.

Comprehensive care delivered by coordinated multidisciplinary teams, incorporating instrumental diagnostics with evidence-based therapeutic interventions, represents the optimal strategy for improving outcomes and quality of life in patients with neurodegenerative diseases affecting swallowing and speech.

## Figures and Tables

**Table 1:** Neural Network Components for Swallowing and Speech Control.

Brain Region	Role in Swallowing	Role in Speech	Key Connectivity
**Cortical**			
Primary Sensorimotor Cortex	Voluntary swallow initiation, oral phase control	Articulator motor control (face, lips, tongue)	Corticobulbar tracts to brainstem nuclei
Insula	Primary integration center, sensory-motor coordination	Articulatory coordination, speech motor control	Connections to sensorimotor cortex, basal ganglia
Cingulate Gyrus	Volitional control, sensory integration	Emotional prosody, motivation	Frontal and parietal networks
Supplementary Motor Area	Movement preparation, sequencing	Speech planning, sequencing	Primary motor cortex, basal ganglia
Broca’s Area	Limited role	Motor speech programming	Arcuate fasciculus to Wernicke’s area
**Subcortical**			
Basal Ganglia	Swallow initiation, timing, amplitude	Speech initiation, amplitude, prosody	Cortico-striato-thalamo- cortical loops
Thalamus	Sensory relay, motor modulation	Motor command relay, feedback	Cortical and cerebellar connections
**Cerebellar**			
Cerebellum	Timing, sequencing, force modulation	Articulation precision, rhythm, prosody	Cerebello-thalamo-cortical pathways
**Brainstem**			
Nucleus Tractus Solitarius	Central pattern generator, sensory integration	Limited role	Vagal and glossopharyngeal afferents
Nucleus Ambiguus	Pharyngeal/laryngeal motor control	Laryngeal control for phonation	Vagal efferents
Hypoglossal Nucleus	Tongue movement	Tongue articulation	Corticobulbar inputs
Facial Nucleus	Oral phase muscles	Lip articulation	Corticobulbar inputs
Trigeminal Motor Nucleus	Jaw movement	Jaw articulation	Corticobulbar inputs

**Table 2:** Disease-Specific Patterns of Multisystem Involvement in Dysphagia and Dysarthria.

Disease	Primary Pathology	Affected Brain Regions	Dysphagia Features	Dysarthria Features
**Parkinson’s Disease**	Alpha-synuclein accumulation, dopaminergic depletion	Substantia nigra, basal ganglia, dorsal motor nucleus vagus, locus coeruleus, raphe nuclei, cortex (late)	Delayed swallow initiation, oral bradykinesia, reduced pharyngeal peristalsis, cricopharyngeal dysfunction, sialorrhea	Hypokinetic: reduced loudness, monotone speech, imprecise articulation, variable rate (10–20% mixed)
**ALS**	Upper and lower motor neuron degeneration	Motor cortex, corticobulbar tracts, brainstem motor nuclei (NA, XII, VII, V)	Tongue weakness, impaired bolus formation, delayed pharyngeal trigger, reduced laryngeal elevation, aspiration	Mixed spastic-flaccid: slow effortful speech, harsh voice, breathy quality, hypernasality
**PSP**	Tau protein accumulation	Brainstem (substantia nigra, superior colliculus), basal ganglia, frontal cortex, cerebellum	Delayed pharyngeal initiation, impaired hyolaryngeal excursion, silent aspiration, early severe dysphagia	Spastic-ataxic: harsh voice, reduced loudness, imprecise articulation, equal stress patterns
**MSA**	Alpha-synuclein in oligodendrocytes	Pontine nuclei, inferior olives, nucleus ambiguus, cerebellum, putamen, autonomic centers	Severe pharyngeal incoordination, laryngeal dysfunction, early aspiration, stridor	Ataxic (MSA-C) or mixed hypokinetic- ataxic: irregular articulatory breakdowns, excess and equal stress

**Table 3:** Comprehensive Diagnostic Tool Summary for Dysphagia and Dysarthria Assessment.

	Specific Tools	Information Provided	Recommended Timing
**Assessment Type**			
Dysphagia	EAT-10, water swallow test, multi- consistency protocol	Risk stratification, aspiration screening	Initial evaluation, every 6 months
Speech	Mayo Clinic classification, FDA- 2, SAND	Dysarthria subtype, severity	Initial evaluation, annually
Cognitive	MoCA, FAB	Cognitive factors affecting function	Initial evaluation, annually
**Instrumental-** **Swallowing**			
Videofluoroscopy (VFS)	Modified barium swallow	All phase dynamics, aspiration/penetration	Gold standard, as indicated
FEES	Fiberoptic endoscopic evaluation of swallowing	Pharyngeal/laryngeal visualization, secretions	When VFS unavailable, follow-up
High-Resolution Manometry	Pharyngeal/esophageal pressures	Pressure profiles, coordination	Suspected esophageal dysfunction
Electromyography	Surface/needle EMG	Muscle activation patterns	Research, botulinum toxin guidance
**Instrumental-** **Speech**			
Acoustic Analysis	Praat, CSL software	F0, jitter, shimmer, VOT, VSA, DDK rates	Objective baseline, treatment monitoring
Aerodynamic Assessment	PAS evaluation	Subglottic pressure, airflow	Suspected respiratory involvement
**Neurophysiological**			
Laryngeal EMG	Needle electrodes	Neuromuscular transmission, reinnervation patterns	Suspected neuropathy
TMS	Transcranial magnetic stimulation	Corticobulbar pathway integrity	Research settings
**Neuroimaging**			
Structural MRI	T1, T2, FLAIR sequences	Atrophy patterns, lesions	Diagnosis confirmation
DTI	Diffusion tensor imaging	White matter tract integrity	Research, selected cases
Functional MRI	Task-based/resting state	Network activation patterns	Research settings
PET	FDG-PET, dopamine imaging	Metabolic activity, dopaminergic function	Differential diagnosis

## Data Availability

No new data were created.

## Abbreviations

The following abbreviations are used in this manuscript:

AAC	Augmentative and Alternative Communication
ALS	Amyotrophic Lateral Sclerosis
BTX	Botulinum Toxin
DDK	Diadochokinetic
DTI	Diffusion Tensor Imaging
EAT-10	Eating Assessment Tool-10
EMG	Electromyography
EMST	Expiratory Muscle Strength Training
F0	Fundamental Frequency
FAB	Frontal Assessment Battery
FDA-2	Frenchay Dysarthria Assessment-2
FEES	Fiberoptic Endoscopic Evaluation of Swallowing
fMRI	Functional Magnetic Resonance Imaging
HRM	High-Resolution Manometry
IDDSI	International Dysphagia Diet Standardisation Initiative
IMST	Inspiratory Muscle Strength Training
LSVT	Lee Silverman Voice Treatment
MoCA	Montreal Cognitive Assessment
MSA	Multiple System Atrophy
NA	Nucleus Ambiguus
NG	Nasogastric
NMES	Neuromuscular Electrical Stimulation
NTS	Nucleus Tractus Solitarius
PAS	Phonatory Aerodynamic System
PD	Parkinson’s Disease
PEG	Percutaneous Endoscopic Gastrostomy
PES	Pharyngeal Electrical Stimulation
PLVT	Pitch Limiting Voice Treatment
PSP	Progressive Supranuclear Palsy
QOL	Quality of Life
rTMS	Repetitive Transcranial Magnetic Stimulation;
RIG	Radiologically Inserted Gastrostomy
SAND	Screening for Aphasia in Neurodegeneration
sEMG	surface Electromyography
tDCS	Transcranial Direct Current Stimulation;
TMS	Transcranial Magnetic Stimulation
UES	Upper Esophageal Sphincter
VFS	Videofluoroscopy
VOT	Voice Onset Time
VSA	Vowel Space Area
